# The Effects of Breeding Protocol in C57BL/6J Mice on Adult Offspring Behaviour

**DOI:** 10.1371/journal.pone.0018152

**Published:** 2011-03-23

**Authors:** Claire J. Foldi, Darryl W. Eyles, John J. McGrath, Thomas H. J. Burne

**Affiliations:** 1 Queensland Brain Institute, The University of Queensland, St Lucia, Queensland, Australia; 2 Queensland Centre for Mental Health Research, The Park Centre for Mental Health, Richlands, Queensland, Australia; 3 Department of Psychiatry, The University of Queensland, St Lucia, Queensland, Australia; Alexander Flemming Biomedical Sciences Research Center, Greece

## Abstract

Animal experiments have demonstrated that a wide range of prenatal exposures can impact on the behaviour of the offspring. However, there is a lack of evidence as to whether the duration of sire exposure could affect such outcomes. We compared two widely used methods for breeding offspring for behavioural studies. The first involved housing male and female C57Bl/6J mice together for a period of time (usually 10–12 days) and checking for pregnancy by the presence of a distended abdomen (Pair-housed; PH). The second involved daily introduction of female breeders to the male homecage followed by daily checks for pregnancy by the presence of vaginal plugs (Time-mated; TM). Male and female offspring were tested at 10 weeks of age on a behavioural test battery including the elevated plus-maze, hole board, light/dark emergence, forced swim test, novelty-suppressed feeding, active avoidance and extinction, tests for nociception and for prepulse inhibition (PPI) of the acoustic startle response. We found that length of sire exposure (LSE) had no significant effects on offspring behaviour, suggesting that the two breeding protocols do not differentially affect the behavioural outcomes of interest. The absence of LSE effects on the selected variables examined does not detract from the relevance of this study. Information regarding the potential influences of breeding protocol is not only absent from the literature, but also likely to be of particular interest to researchers studying the influence of prenatal manipulations on adult behaviour.

## Introduction

Accumulating epidemiological evidence suggests that adverse early life events, such as childhood trauma and neglect, can affect emotional behaviour and risk for depression, anxiety disorders and substance abuse [1], [2], [3], [4]. Thus, it is important to understand how events in early life can contribute to the development of individual differences in stress vulnerability. Animal studies have clearly established that the prenatal and early postnatal rearing environment influences adult behavioural responses to acute stress [5], [6], [7]. There is robust evidence showing that maternal stress during gestation in mice, such as restraint stress [8], [9], [10] and foot shock stress [11], affects prenatal offspring development and subsequent adult behavioural responses to stress. Furthermore, enrichment of the prenatal environment in mice has been demonstrated to alter locomotor activity and the amount of hippocampal cell proliferation in offspring [12]. Therefore, it is reasonable to assume that many environmental factors during gestation will affect early postnatal development and lead to altered behaviour in adult offspring. In mammals, the intense prenatal and postnatal investment by the mother and the rarity of bi-parental care has directed most research to date to the influence of *maternal factors*. However, it is also feasible that the behaviour of the sire (i.e. *paternal factors*) could alter either prenatal maternal stress levels or even post-natal care, which in both cases could influence subsequent behaviour of the offspring. While one study has attempted to explore the strength of paternal-offspring behavioural correlations versus the length of sire exposure (LSE) in Balb/cJ mice [13], to the best of our knowledge no studies have specifically examined the influence of prenatal LSE on offspring behaviour.

The duration of prenatal sire exposure could impact on maternal stress via several mechanisms. The behaviour of the sire may, for example, change hormone levels in the dams [14] or alter the maternal environment through variation in male phenotypic qualities [15]. Female mate preference may depend on such phenotypic qualities and has been assessed using a four-chamber preference task. Curiously, when females mated with a preferred male, they gave birth to offspring that demonstrated stronger adaptive behaviours such as social dominance, nest building and avoidance behaviour, compared to the offspring of females mated with non-preferred males [16]. While most breeding facilities for laboratory animals do not offer female mate preference, behavioural characteristics of sires that influence mate preference could nonetheless affect maternal investment in offspring. If this is the case, it is likely that sire effects on offspring outcomes would be greater if they were exposed to pregnant females for a longer duration post-conception. Of course, it would be expected that some features of offspring behaviour would reflect paternal traits via genetic variation (especially in out-bred strains). However, it is also feasible that variables such as short versus prolonged prenatal sire exposure could influence maternal stress levels, and thus indirectly impact on offspring behaviour.

We are interested in developing rodent models in order to explore epidemiological findings linking prenatal manipulations with a range of adverse health outcomes in offspring. In particular, we have previously demonstrated that the offspring of older (12–18 month-old) C57BL/6J mouse sires have changes in exploratory and anxiety-related behaviours and an altered trajectory of cortical development [17]. This previous study utilised a breeding protocol that involved housing male and female mice together for a period of time (10–12 days) and checking for pregnancy by the presence of a distended abdomen (Pair-housed; PH). This is the most common method used to breed animals for adult behaviour testing. By using this protocol, however, we were unable to determine whether the alterations in offspring development reported as resulting from differences in paternal age were associated with genetic effects of age, via mutations in the sperm, or behavioural effects of age, via dam-sire interactions. As part of a wider research program, we wish to explore if the nature of the behavioural outcomes we found could be influenced by LSE, and in particular, could be influenced by one of two widely used breeding protocols for mice. The first is the PH strategy used in the above study [17]. The second involved daily introduction of female breeders to the male housing followed by daily checks for pregnancy by the presence of vaginal plugs (Time-mated; TM). This is the most common method used in developmental studies where precise embryo ages are required. The abundance of literature on maternal stress and its effects on anxiety-related behaviour in offspring is convincing, and LSE could potentially modulate these effects. While it would be of interest to directly investigate stress levels by measuring cortisol or other hypothalamic-pituitary-adrenal (HPA) axis outcomes in sire-exposed dams, our focus here is on the influence of early life events on offspring behaviour. Therefore, our primary aim was to determine whether LSE alone affected a range of behavioural domains, with an emphasis on anxiety-related behaviour in adult offspring. In addition, we aimed to examine the effects of sire exposure on learning and sensorimotor gating, as these are frequently used behavioural paradigms in mouse models of neuropsychiatric disorders.

## Results

Firstly, Breeding Protocol had no significant effects on major reproductive outcomes (see Table 1). Litter size (*F*_1,15_ = 0.45, *p = *0.51), litter maintenance (*F*_1,15_ = 0.55, *p = *0.47) and the sex ratio of litters (Male; *F*_1,15_ = 0.60, *p = *0.45, Female; *F*_1,15_ = 0.01, *p = *0.89) were unchanged. Further, adult offspring body weights were equivalent for PH and TM breeding dyads (Male; *t*_33_ = 0.84, *p* = 0.41, Female; *t*_40_ = −0.43, *p* = 0.67). There was a significant effect of Breeding Protocol on days to conception (*F*_1,15_ = 5.01, *p = *0.04), such that PH dyads took longer to achieve mating success than did TM dyads.

**Table 1:** Mean ± S.E.M. values for reproductive outcomes in Time-mated and Pair-housed breeding dyads.

		*Breeding Group*					
Reproductive Outcome	Measurement	Time-mated			Pair-housed		
Mating success	**Days to conception**	2.94	±	0.59	4.57	±	1.00
Litter size	**Pups born (n)**	6.00	±	0.32	5.46	±	0.42
Litter maintenance	**Pups survived (n)**	2.94	±	0.71	3.57	±	0.82
Sex ratio	**Male pups (n)**	1.19	±	0.32	1.64	±	0.48
	**Female pups (n)**	1.87	±	0.46	1.93	±	0.58
Body weights	**Adult male offspring (g)**	26.13	±	0.45	25.66	±	0.28
	**Adult female offspring (g)**	20.12	±	0.24	20.28	±	0.28
	***Total litters (n)***	**15**			**13**		

There were no significant effects of Breeding Protocol on any of the primary measures of anxiety in this study (see Table 2). Offspring from PH and TM parental dyads spent a similar percentage of time on the open arms of the EPM (*F*_1,73_ = 1.10, *p = *0.29) and in the centre of the hole board apparatus (*F*_1,73_ = 3.77, *p = *0.06). The latencies to emerge from the dark compartment of the light/dark test (*F*_1,73_ = 0.42, *p = *0.84) and to approach the food to eat in the novelty-suppressed feeding test (Day 1; *F*_1,73_ = 0.20, *p = *0.66, Day 2; *F*_1,73_ = 0.95, *p = *0.33) were also not significantly altered by Breeding Protocol.

**Table 2:** Mean ± S.E.M. values for behavioural outcomes in all domains assessed for male and female Time-mated and Pair-housed offspring.

			*Male*						*Female*					
Domain	Test	Parameter measured	Time-mated			Pair-housed			Time-mated			Pair-housed		
Anxiety	EPM	**Duration on open arms (%)**	13.03	±	1.43	10.59	±	1.23	13.53	±	1.40	12.83	±	1.75
	Holeboard	**Duration in centre (s)**	24.11	±	2.39	29.22	±	5.47	30.82	±	3.96	32.69	±	5.01
	Light/Dark	**Latency to emerge (s)**	13.10	±	3.00	12.20	±	3.50	27.90	±	8.70	25.90	±	9.30
	NSF Day1	**Latency to eat (min)**	1.88	±	0.33	2.14	±	0.61	2.75	±	0.59	2.78	±	0.65
	NSF Day2	**Latency to eat (min)**	0.32	±	0.07	0.63	±	0.31	1.17	±	0.42	1.34	±	0.42
Locomotion	EPM	**Distance travelled (cm)**	1943.03	±	56.28	1797.12	±	118.47	2054.99	±	82.09	2250.73	±	105.18
	Holeboard	**Distance travelled (cm)**	2449.44	±	189.40	2600.64	±	155.54	2752.51	±	175.37	2963.10	±	174.12
Exploration	EPM	**Head dipping (counts/10 min)**	4.28	±	0.74	5.18	±	1.09	4.32	±	0.57	6.30	±	1.12
	Holeboard	**Head dipping (counts/10 min)**	7.17	±	0.79	8.59	±	1.02	7.14	±	0.95	7.85	±	0.49
	EPM	**Rearing (counts/10 min)**	21.78	±	1.82	22.82	±	2.42	24.96	±	2.23	25.80	±	2.75
	Holeboard	**Rearing (counts/10 min)**	27.33	±	3.51	30.65	±	2.40	32.46	±	3.08	34.60	±	2.60
Learned Helplessness	FST													
	Day 1	**Immobile time (%)**	37.93	±	3.30	42.63	±	2.83	40.69	±	2.28	39.01	±	3.34
	Day 2	**Immobile time (%)**	43.11	±	3.05	47.90	±	3.69	38.12	±	3.26	42.37	±	1.86
Avoidance Learning	Day 1	**CAR (%)**	52.43	±	3.99	54.27	±	3.62	56.76	±	2.98	59.92	±	3.42
	Day 2	**CAR (%)**	89.57	±	1.36	90.47	±	1.52	92.71	±	0.90	93.23	±	0.80
	Day 3	**CAR (%)**	56.50	±	9.02	50.67	±	9.08	60.00	±	9.68	68.23	±	6.89
Nociception	Tailflick	**Latency to flick (s)**	5.22	±	0.67	5.69	±	0.85	5.58	±	0.74	5.00	±	0.60
	Hotplate	**Latency to lick hindpaw (s)**	17.21	±	1.15	20.95	±	1.49	17.17	±	0.76	17.53	±	1.21
PPI of the ASR														
74 dB	Day 1	**PPI (%)**	9.86	±	9.76	7.50	±	13.69	19.33	±	5.39	2.72	±	8.79
	Day 2	**PPI (%)**	-3.06	±	8.32	5.33	±	10.07	5.29	±	11.36	7.09	±	9.37
78 dB	Day 1	**PPI (%)**	33.77	±	8.62	26.56	±	11.97	26.20	±	6.09	28.79	±	5.50
	Day 2	**PPI (%)**	21.21	±	9.84	32.40	±	6.62	23.20	±	8.22	27.36	±	9.09
86 dB	Day 1	**PPI (%)**	45.12	±	7.51	48.60	±	8.18	40.61	±	3.69	43.11	±	5.79
	Day 2	**PPI (%)**	43.43	±	8.31	52.49	±	6.07	36.89	±	6.86	45.24	±	7.27
		***n***	**18**			**17**			**22**			**20**		

PH and TM offspring also showed no significant differences on other standard measures tested in this battery, including locomotion (EPM; *F*_1,73_ = 0.71, *p = *0.79, hole board; *F*_1,73_ = 1.05, *p = *0.31), exploration by head dipping (EPM; *F*_1,73_ = 0.49, *p = *0.49, hole board; *F*_1,73_ = 1.62, *p = *0.21), rearing (EPM; *F*_1,73_ = 0.16, *p = *0.69, hole board; *F*_1,73_ = 0.84, *p = *0.36) and learned helplessness on the forced swim test (Day 1; *F*_1,73_ = 0.26, *p = *0.61, Day 2; *F*_1,73_ = 2.23, *p = *0.14).

Repeated measures ANOVA revealed no significant effects of Breeding Protocol on the percentage of CAR in either the acquisition (Day 1; *F*_1,55_ = 0.51, *p = *0.48), maintenance (Day 2; *F*_1,55_ = 0.32, *p = *0.58), or extinction (Day 3; *F*_1,55_ = 0.02, *p = *0.89) of active avoidance learning. Similarly, tests of nociception showed no significant alteration in pain threshold between PH and TM mice on either the hotplate (*F*_1,59_ = 3.27, *p = *0.08) or tail flick (*F*_1,59_ = 0.01, *p = *0.94) tests.

Finally, there was no significant effect of Breeding Protocol on PPI at any of the prepulse intensities investigated; 74 dB (Day 1; *F*_1,35_ = 0.79, *p = *0.38, Day 2; *F*_1,35_ = 0.23, *p = *0.64), 78 dB (Day 1; *F*_1,35_ = 0.07, *p = *0.80, Day 2; *F*_1,35_ = 0.76, *p = *0.39) or 86 dB (Day 1; *F*_1,35_ = 0.19, *p = *0.66, Day 2; *F*_1,35_ = 1.39, *p = *0.25). Table 2 displays the mean ± S.E.M. on all primary measures assessed in this study for male and female TM and PH offspring.

There was a significant main effect of Sex on locomotion in the EPM, with a greater distance travelled by female (2148.00±66.95 cm) compared to male mice (1872.16±64.65 cm) on this test (*F*_1,73_ = 9.19, *p* = 0.003). In addition, female mice took longer to approach the food to eat (1.25±0.30 s) than did males (0.47±0.16 s) on Day 2 of the novelty-suppressed feeding test (*F*_1,73_ = 4.71, *p* = 0.03). Finally, females exhibited a greater percentage of CAR (92.93±0.61%) than male mice (90.03±1.10%) on Day 2 of the avoidance learning schedule (*F*_1,73_ = 5.42, *p* = 0.02).

There were no significant main effects of Sex on any other measures examined in this study and there were no significant Sex x Breeding Protocol interactions. For example, anxiety-related behaviour on the EPM (Sex; *F*_1,73_ = 0.84, *p* = 0.36, Interaction; *F*_1,73_ = 0.34, *p* = 0.56), exploration by head dipping on the hole board (Sex; *F*_1,73_ = 0.21, *p* = 0.65, Interaction; *F*_1,73_ = 0.18, *p* = 0.68), latency to emerge from the dark compartment of the light/dark test (Sex; *F*_1,73_ = 3.85, *p* = 0.09, Interaction; *F*_1,73_ = 0.005, *p* = 0.94), and percent time immobile on the forced swim test on either Day 1 (Sex; *F*_1,73_ = 0.02, *p* = 0.89, Interaction; *F*_1,73_ = 1.16, *p* = 0.29) or Day 2 (Sex; *F*_1,73_ = 3.01, *p* = 0.10, Interaction; *F*_1,73_ = 0.01, *p* = 0.93) were unchanged.

## Discussion

The length of sire exposure (LSE) to the maternal environment had no significant effects on offspring behaviour in a range of commonly assessed behavioural domains in C57Bl/6J mice. The data from this study suggest that, compared to a restricted schedule of time-mating, twelve days of prenatal sire exposure is not sufficient to alter adult offspring behaviour. This result is of great importance to researchers interested in the effects of prenatal manipulations, for example, in animal models of human disorders. There are no published reports of effects of prenatal sire exposure on offspring behaviour with which to compare these results, however, behavioural alterations have been demonstrated in offspring exposed to sires postnatally [18]. While there were no significant Sex x Breeding Protocol interactions, there were some main effects of Sex found in this study, consistent with reports of female mice being more active than males [19]. Although it has been suggested that mate selection mediates some paternal effects [16], all mice used in this study were obtained from the same source, at the same age, and are therefore likely to have equivalent phenotypic quality.

There was a significant effect of Breeding Protocol on mating success, determined by days to conception, in that PH dyads took longer to achieve mating success than did TM dyads. This is likely to be an artefact of the breeding method itself. PH females were not separated on a daily basis, thereby increasing the chance that a positive plug would remain undetected. For example, if mating occurred in PH dyads shortly after checks were made, the vaginal plug may not be detectable the following morning (at the next check). Interestingly, this study demonstrated that independent of Breeding Protocol, there was a sizeable discrepancy between litter size (i.e. number of pups born) and litter maintenance (i.e. number of pups survived), indicating that all litters were subjected to early death via dam-mediated mechanisms such as cannibalisation or neglect. One possible explanation for this is that all dams used in this study were nulliparous, that is, they had not littered previously. They were chosen as such to control for age and differences in maternal experience. Litter survival is shown to improve significantly with experience of maternal behaviour and increasing parity in oldfield mice [20], therefore lack of experience with parturition may explain the discrepancy in this study. In addition, the animal facility as a whole has been experiencing difficulty with cannibalisation due to a number of factors including high-pitched noise from the air pressure control, external noise from infrastructure building nearby on the campus and the use of individually ventilated cages.

The body of evidence implicating maternal stress in determining offspring behavioural responses to stress is convincing. However, most studies in this field demonstrate such effects when the stressor is introduced or continues throughout the later stages of gestation, particularly the final gestational week [9], [10], [11]. While this is true for rats, it is known that maternal stress early in pregnancy in mice can significantly impact embryonic development, as early as seven days post-conception [21]. Thus, sire exposure in mice during this early developmental period was not sufficiently stressful to alter the maternal environment. Although not in keeping with routine animal house breeding protocols, it would be of interest to extend an LSE protocol into the later period of gestation to determine the precise stages of development that may be affected by LSE [22]. Maternal stress reactivity may in fact be equally disrupted by both the continuous sire exposure (PH) and daily handling (TM) protocols, since handling of pregnant rodents is known to alter offspring behaviour [23], [24]. Unfortunately no measure of maternal stress (i.e. HPA function) could be undertaken in this study due to the invasive nature of such studies. Thus, whether PH or TM procedures alter maternal stress responsivity remains unknown. Another caveat to this study was that the precise timing of conception in the PH group was not known. Therefore, there is likely to be variability in the number of days post-conception in which the dams were exposed to sires. The behavioural results may be confounded by some post-conception versus peri-conception LSE.

There is good epidemiological evidence showing that the offspring of older fathers have an increased risk of schizophrenia [25], [26], [27], autism [28], [29], [30] and impaired cognition in childhood [31] and early adolescence [32]. The mechanisms underpinning these associations remain unclear, but could include additional mutational loads in the germ line of older fathers [33], [34], [35] and/or epigenetic changes in the sperm of older fathers [36], [37]. In response to these research questions, several groups have developed rodent models to explore the association between paternal age and offspring behaviour [17], [38], [39]. Because it is known that the behaviour of rodents changes with age [40], [41], and that fertility and general mating success decline in older sires [42], it is feasible that these factors could impact on protocols such as pair-housing that result in longer peri-conceptual sire exposure. The results of the current study suggest that the duration of sire exposure does not impact on the outcomes of interest, however TM breeding strategies would reduce the impact of this potential confounding variable.

Although there is a wealth of research exploring the association between maternal factors during the perinatal period and their effects on the behaviour of adult offspring, the direct or indirect influence of sire behaviour during the perinatal period has largely been ignored. With the growing body of research linking advanced paternal age with altered offspring behaviour [17], [31], [38], [39], [43], and with recently published studies now linking paternal diet with increased risk of disease in offspring (e.g. diabetes) [44], greater focus on the influence of paternally-mediated factors on offspring health is warranted. Apart from the influence of the environment on paternal germ-line development, it is conceivable that paternal behaviour during the periconceptual period could impact on maternal stress levels, and subsequently on the offspring behaviour. Our study indicates that two commonly used breeding programs do not impact on the range of behavioural outcomes selected for this study. While LSE was not associated with the outcomes of interest, it is still feasible that periconceputal behaviour of the sire (regardless of LSE) may influence maternal stress. The research community needs to remain alert for potential confounding factors that may influence behavioural outcomes in experiments that manipulate paternal factors.

## Materials and Methods

### Ethics Statement

All procedures were performed with approval from the University of Queensland Animal Ethics Committee, under the National Health and Medical Research Council of Australia guidelines.

### Animals and housing

All mice were sourced from the University of Queensland animal facility. Fifteen (*n* = 8 PH, *n* = 7 TM) 10 week-old C57BL/6J mouse pairs were mated to obtain offspring for this study. All males were exposed to a training female for the first time 7 days prior to commencement of the experimental breeding. This was used to control for amount of sexual experience across sires and to prevent breeding effects related to unfamiliarity with female mice. After this period of socialisation training, a naive nulliparous female mouse was placed into each sire cage and either left for a period of 12 continuous days (pair-housed; PH), after which pregnancy was detected by visual inspection of a distended abdomen, or introduced at 1600 h each evening and examined by visual inspection for vaginal plugs at 0900 h the following morning (time-mated; TM). If no plug was observed, females in the TM condition were re-housed in groups and this process was repeated for a maximum of five days. TM females were always re-mated with the same sire. Once breeding protocols were complete, all females were housed separately in individually ventilated OptiMice cages at the Queensland Brain Institute animal facility, University of Queensland St Lucia campus. The facility operated on a 12 h light/dark schedule (lights on at 0700 h) and animals had access to standard mouse food (Feeder and Grower diet, Specialty Feeds, WA) and water *ad libitum*. Offspring from both PH (*n = *37; 17 male, 20 female) and TM (*n = *40; 18 male, 22 female) conditions were weaned at 4 weeks of age and housed in same-sex groups of 3–5.

### Procedure

Behavioural phenotyping began when offspring were 10 weeks of age and tests were conducted on separate and consecutive days in the following order; elevated plus-maze (EPM), hole board, light/dark emergence, 2-day forced swim test, 2-day novelty-suppressed feeding. The order of testing was such that the tests most sensitive to handling were performed first and those most stressful performed last. Following one week of free feeding, animals were tested on a 3-day active avoidance and extinction protocol, tests for nociception and for prepulse inhibition (PPI) of the acoustic startle response. All behavioural observations were made blind to experimental condition (Breeding Protocol) and, where required, recorded from a central overhead camera, which was attached to computerised tracking and event-recording software, EthoVision® ver.3.1 (Noldus, Netherlands). Mice were always acclimated to the testing room for 1 hr prior to test commencement and all arenas and apparatus were cleaned between trials with 20% ethanol.

### Elevated Plus-maze (EPM)

The EPM was used to obtain a measure of anxiety-related behaviour [45] as well as to assess exploration and locomotion [46]. The EPM was made with opaque grey acrylic and consisted of two opposing pairs of arms, one open (5×30 cm) and one closed (5×30×30 cm high) extending from a central platform (5×5 cm) that was positioned 50 cm above the ground. Mice were placed on the central platform facing one of the open arms. During each 10 min trial, the distance moved was measured, as was the frequency, duration and latency of ethologically derived behaviours including rearing, stretching, grooming and head-dipping. The percentage of time that animals spent on the open arms of the maze, relative to closed arms, was used as the primary measure of anxiety-related behaviour.

### Hole Board

The hole board test was used to assess exploration and locomotion [46]. The hole board consisted of an opaque white acrylic box (30×30×30 cm) with a raised (2.5 cm) floor insert containing four holes (2.5 cm diameter, 5.3 cm from each corner). Mice were placed individually in the centre of the hole board and each trial lasted 10 min. Parameters measured included distance travelled and ethologically derived behaviours including rearing, stretching, grooming and head-dipping. Frequency and duration of head dipping was used as the primary measure of exploration. Duration spent in the centre of the arena was used as the primary measure of anxiety in this test [47].

### Light/Dark Emergence

Eight individual activity monitors (27.9×27.9 cm) with three 16 beam infrared arrays (MED Associates, Inc., Georgia, VT) were used for this test, each containing a darkened acrylic insert that was penetrable by infrared light and sheltered half the arena. Latency to enter the light half of the arena, through an opening in the dark insert, was the primary measure of anxiety in this test.

### Two-day Forced Swim Test

Learned helplessness was assessed using the forced swim test [48]. In this test, a clear plastic cylinder (20 cm high × 13 cm diameter) was filled to three-quarter capacity with tap water at 25 ±1°C. Animals were placed into the water from approximately 5 cm above the cylinder's rim. The test duration was 10 min on the first day and 5 min on the second, conducted 24 h apart. Swimming activity was scored using the mobility threshold settings within the EthoVision software by measuring the percentage change in area of the tracked object from one sample to the next. Activity was defined as either immobile (0–20%) or strongly mobile (60–100%). Unlike the test in rats, in the Porsolt forced swim paradigm for mice it is recommended to analyse immobility time between 3 and 6 min on Day 1 of the test. Day 2 is used only to assess task acquisition deficits [48].

### Two-day Novelty-suppressed Feeding

Following the forced swim test, mice were food deprived for 24 h before being placed in an opaque white acrylic box (30×30×30 cm) that contained a food pellet chip (Feeder and Grower diet, Specialty Feeds, WA) placed on a 5×5 cm piece of filter paper. The test duration was 10 min on the first day and 10 min on the second, conducted 24 h apart. After testing on Day 1, mice were given approximately 1 g of food, which was sufficient to maintain their weight above 85% of free feeding body weight. Latency to pick up the food chip to eat was used as the primary measure of anxiety on this test.

### Active Avoidance Learning and Extinction

Avoidance learning is a classical conditioning paradigm in which a conditioned response is achieved after multiple pairings of neutral conditioned stimuli (CS) and aversive unconditioned stimuli (US). Active avoidance was conducted in automated two-way shuttle boxes (Gemini Avoidance System, San Diego Instruments, SD USA), modified from a previously reported method [49]. The interior was divided into two sound attenuated chambers (24×20×17 cm), with a stainless steel gate providing access between chambers. Eight infrared photobeams in each chamber were used to detect movement. The floor consisted of evenly spaced stainless steel bars attached to a current device that would deliver a scrambled electric shock at 0.3 mA (the US). A cue light (15 W bulb) and a tone from a speaker situated in the ceiling of each chamber were used in combination to deliver the CS.

Each mouse was placed in the left hand chamber of the two-way shuttle box and the internal gate was closed to allow the mouse to habituate to one chamber for 5 min. All mice were then subject to 100 trials of avoidance learning. Each trial began when the CS was presented and the internal gate opened. After 5 s, the US was delivered through the bars of the floor. If the mouse moved into the opposite chamber during the CS, the CS was terminated, the gate closed and no US was delivered (conditioned avoidance response; CAR). If the mouse moved into the opposite chamber during the US, both the CS and US were terminated and the gate was closed (escape reaction). If the mouse failed to move to the opposite chamber after 2 s of US presentation (hence after a total of 7 s), the trial was terminated and the gate closed (no response). The Gemini software automatically recorded the number of CAR, escapes and no responses made as well as the latency to respond by each mouse during the session. Acquisition (Day 1) and maintenance (Day 2) of the conditioned response was followed by extinction (Day 3), in which all parameters were the same except no US was presented in any of the trials.

### Hot Plate and Tail Flick Tests

In order to eliminate effects of pain threshold (nociception) from differences in avoidance learning from foot shock, two tests of nociception were conducted. For the hot plate test, an automated hot plate (Harvard Instruments, USA) was heated to 55°C and each mouse was placed individually on the plate, which was surrounded by a clear Perspex cylinder to prevent escape. The latency for the mouse to lick its hind paw in response to the heat was used as the measure of nociception. For the tail flick test, mice were restrained in a tube with only it's hind legs and tail protruding. A high intensity light (150 W) at 30% maximum intensity was focused on the dorsal surface of the tail, using a standard tail flick apparatus (Harvard Instruments, USA). An infrared beam recorded latency of tail movement at which point the light was terminated. Each mouse was tested with three trials and the mean latency of these was used in the analysis as a measure of nociception.

### Prepulse Inhibition (PPI) of Acoustic Startle

The PPI paradigm assessed sensorimotor gating and was based on a previous method [50]. PPI refers to the inhibition of the reflexive response to a powerful auditory stimulus (pulse) when this stimulus is preceded by a weaker stimulus (prepulse) [51]. Testing was conducted in sound-attenuating chambers (SR-Lab, San Diego Instruments), each containing a clear Plexiglas cylinder (12 cm long × 5 cm diameter) mounted on a Plexiglas platform with a piezoelectric transducer attached below; to measure startle amplitude.

Computer-controlled startling pulses were delivered through a speaker placed 24 cm above the cylinder. A 5 min acclimation period commenced the startle session, in which baseline movement was assessed at a background noise level of 70 dB. The session then presented a pseudo-randomised order of 26 trial types, each 5 times, which consisted of either the sound pulse alone or the sound pulse preceded by weaker prepulse. Startle trial sound pulses (30 ms duration) were delivered at a range of intensities (80, 90, 100, 110, 120 dB) and 3 blocks of 110 dB pulses were presented at the beginning, middle and end of the session to measure within-session habituation. Prepulse trials comprised three prepulse intensities (74, 78 and 86 dB) at a range of prepulse-pulse intervals (8, 16, 32, 64, 128 and 256 ms) preceding a 120 dB pulse. The median value for each trial type was used in the analysis. The percentage PPI was calculated as [(startle amplitude on pulse alone trials – startle amplitude on prepulse trials)/startle amplitude on pulse alone trials] x 100.

### Statistical Analyses

Results were analysed for statistical significance using the SPSS software package (ver. 17, SPSS Inc., Chicago, Illinois). Multivariate analysis of variance (MANOVA) were used to assess main effects of Breeding Protocol and Sex, and their interactions. Repeated measures ANOVA were used to assess within test effects, for PPI at three levels of prepulse intensity (74, 78 and 86 dB) and six levels of pulse-to-prepulse interval (8, 16, 32, 64, 128 and 256 ms). Percent free-feeding body weight was fitted as a covariate when examining effects on novelty-suppressed feeding. All data are reported as mean ± standard error of the mean (S.E.M.). In all tests, *p*<0.05 (two-tailed) indicated statistical significance.
